# Effects of selenium supplementation on glycemic control markers in healthy rodents: A systematic review protocol

**DOI:** 10.1371/journal.pone.0261985

**Published:** 2022-04-07

**Authors:** Rannapaula Lawrynhuk Urbano Ferreira, Ângela Waleska Freire de Sousa, Antonio Gouveia Oliveira, Adriana Augusto de Rezende, Ricardo Ney Cobucci, Lucia Fatima Campos Pedrosa

**Affiliations:** 1 Postgraduate Program in Nutrition, Federal University of Rio Grande do Norte, Natal, RN, Brazil; 2 Department of Pharmacy, Federal University of Rio Grande do Norte, Natal, RN, Brazil; 3 Department of Clinical and Toxicological Analyses, Federal University of Rio Grande do Norte, Natal, RN, Brazil; 4 Graduate Program of Biotechnology–Universidade Potiguar (UnP), Natal, RN, Brazil; 5 Graduate Program in Sciences Applied to Women’s Health, Maternidade Escola Januário Cicco (MEJC / EBSERH), Federal University of Rio Grande do Norte, Natal, Brazil; 6 Department of Nutrition, Federal University of Rio Grande do Norte, Natal, RN, Brazil; Universitat de Valencia, SPAIN

## Abstract

**Background:**

*In vivo* and *in vitro* studies have shown that Se has an insulin-mimetic action associated with its antioxidant activity. Other studies, in turn, suggest that high Se doses and high selenoprotein expression interfere with insulin signaling. This study aims to evaluate the effects of Se supplementation on glycemic control markers in healthy rodents.

**Methods:**

The protocol was developed according to the Preferred Reporting Items for Systematic Review and Metaanalysis Protocol (PRISMA-P) and was published in the International Prospective Register of Systematic Reviews database (PROSPERO; CRD4202121201142019119181). Experimental, randomized, or non-randomized studies of healthy rodents models will be included. All forms of supplemented Se will be considered, including organic, inorganic, and synthetic compounds, selenium-enriched yeasts, zerovalent Se nanoparticles, and selenized polysaccharides. Fasting blood glucose will be considered the primary outcome. Homeostatic model assessment, plasma and erythrocyte Se concentration, GPX activity, SELENOP concentration, and other Se biomarkers will be considered secondary outcomes. EMBASE, Scopus, Pubmed/Medline, Web of Science, and CINAHL will be searched for articles published with no language restrictions. Two reviewers will independently conduct the search and selection of articles, data extraction, and quality analysis. The risk of bias and methodological quality analyses of the included studies will be performed using the Systematic Review Center for Laboratory Animal Experimentation (SYRCLE) and Collaborative Approach to Meta-Analysis and Review (CAMARADES) tools, respectively. The results will be presented as a narrative synthesis according to the Synthesis Without Meta-analysis (SWiM) Reporting Guideline. Meta-analyses will be conducted where appropriate using random-effects models.

**Discussion:**

The review may clarify the interaction between different forms of supplemented Se and glycemic control in rodents models. The results will provide evidence that will help select doses and forms of Se to administer in clinical trials while according to impact on the glycemic control while elucidating mechanisms of Se metabolism.

## Introduction

Selenium (Se) exerts its antioxidant function through selenoproteins acting in thyroid hormone metabolism and cell growth regulation and protection. Due to its insulin-like effects, Se has been associated with protection against abnormalities in glucose metabolism [1]. Nevertheless, primary epidemiological studies implicate supranutritional Se intake as a potential factor in the development of type 2 diabetes mellitus (T2DM) [2, 3].

The Selenium and Vitamin E Cancer Prevention Trial (SELECT) [2] demonstrated that the incidence of newly diagnosed T2DM increased among participants supplemented with 200 μg of selenomethionine daily for more than 7 years. Nutritional Prevention of Cancer (NPC) data [3] also showed an increased risk of T2DM in participants with plasma Se concentrations in the top tertile at baseline. However, in a randomized clinical trial, daily Se supplementation (200 μg/day of selenized yeast) for 3 years did not affect insulin sensitivity [4]. These findings suggest that different doses and forms of Se may interfere with the association between Se and diabetogenic effects.

A review of animal models concluded that a high Se intake, cooperatively or independently, may contribute to the pro-diabetic potential of Se [5–9]. Supranutritional Se doses also triggered a decrease in insulin sensitivity, causing hyperinsulinemia, insulin resistance, and glucose intolerance due to the overexpression of glutathione peroxidase (GPx) [5, 10]. However, subsequent experimental studies indicate that supplementation with selenite and selenate in diabetic db/db mice and Wistar rats stimulates the gene expression of β cells and increases glucose uptake by peripheral tissues supporting the insulin-mimetic function performed by Se [11–13].

Considering that high blood glucose is third among factors most associated with premature mortality [14, 15] and the divergent findings of preclinical and clinical studies on the effect of Se on glucose metabolism, evidence on mechanisms that associate Se with glycemic control must be sought.

Thus, this systematic review will aim to evaluate the effects of Se supplementation on glycemic control markers in healthy rodents.

## Methods/Design

The systematic review protocol follows the recommendations of the Preferred Reporting Items for Systematic Review and Meta-analysis Protocol (PRISMA-P) [16], and to ensure the quality of the protocol, the PRISMA-P checklist was completed (S1 Checklist). During the review, changes in the protocol may be made, which will be duly justified and published through the International Prospective Register of Systematic Reviews (PROSPERO) database. The protocol was published on PROSPERO (registration number CRD4202121201142019119181)

### Review question

The following review question was established: “Does Se supplementation affect glycemic control markers in healthy rodents?” The question was formulated based on the acronym PICOS, which considers P, population; I, intervention; C, comparison; O, outcome; S, study design (Table 1).

**Table 1:** Definition of the structured review question in the form of the acronym PICOS.

Description	Abbreviation	Elements
**Population**	P	Healthy rodent animal models
**Intervention**	I	Selenium supplementation
**Comparison**	C	Control—not supplemented
**Outcome**	O	Glycemic control markers and selenium biomarkers
**Study design**	S	Experimental studies with control group

### Eligibility criteria

#### Animal model of interest

Studies with experimental animal models of class Mammalia (mammals), order Rodentia (rodents) comprising healthy individuals of both sexes subjected to Se supplementation will be included. Studies with animal models of other designations and/or with induced diseases or clinical conditions of any nature will be excluded, and so will be non-experimental models (wild or pet animals), pregnant, weaned, and pubescent animal models.

#### Intervention of interest

All forms of supplemented Se will be considered, including organic, inorganic, and synthetic compounds, selenium-enriched yeasts, zerovalent Se nanoparticles (SeNPs), and selenized polysaccharides (SPs). All supplementation dosages and administration routes will be considered, including gavage, intraperitoneal, orogastric or oral, simulating the natural route of the diet. Rodents subjected to Se supplementation combined with other micronutrients (vitamins or minerals) will be excluded.

#### Comparison of interest

Experimental animal models of class Mammalia, order Rodentia comprising healthy animals of both sexes that have been exposed to basal or standard laboratory diets without supplementation of selenium for weight maintenance will be considered a control.

#### Outcome measures

The fasting plasma glucose (FPG) concentration will be considered the primary outcome. Secondary outcomes include other glycemic markers and Se biomarkers (Table 2). All markers unrelated to Se status and glycemic control will be excluded.

**Table 2:** Primary and secondary outcomes to be measured.

Markers		Outcomes
**Glycemic control**	FPG (mg/dL)	Primary
	OGTT (mg/dL), HbA1c (mmol/mol), serum insulin concentration (pUI/mL or pmol/L), HOMA-IR, HOMA-B and QUICKI.	Secundary
**Selenium biomarkers**	GPX expression and activity (U/g Hb), SELENOP (mg/L), plasma selenium concentration (ng/ml or μg/L or μg/mL), erythrocyte selenium concentration (μg/gHb or μg/L) and tissues selenium concentration (μg/g or μg/kg). Selenium urine concentration (μg/L or μg/mL)	

Abbreviations: FPG, Fasting plasma glucose; OGTT, Oral glucose tolerance test; HbA1c, Glycated hemoglobin A1c; HOMA-IR, Homeostatic Model Assessment of Insulin Resistance; HOMA-B, Homeostatic model assessment of β cell function; QUICKI, Quantitative Insulin sensitivity Check Index; GPx, glutathione peroxidase; SELENOP, selenoprotein P.

#### Study designs to be included

All experimental studies with a control group, randomized or non-randomized, will be included. Case reports, reviews, dissertations, theses, letters, and editorials will be excluded, as well as, in vitro, in silico, ex vivo, and before-after studies without a control group.

### Search strategy

The review authors will search the MEDLINE database through PubMed, Web of Science, Embase, CINAHL, and Scopus.

The search strategy will use a combination of medical subject headings (MeSH) and “entry terms.” The search terms will be divided into three components, i.e., population, intervention, and outcomes components. These will include the terms “animal model,” “Rodentia,” “selenium supplementation,” “selenium,” “selenite,” “blood sugar,” “glucose tolerance test,” “insulin,” “insulin response,” “glucose metabolism,” “glutathione peroxidase,” “GPx expression,” and “selenoprotein P.” However, during the search, other terms may be included or changed according to the characteristics of the databases.

Search filters for identifying animal studies in PubMed and Embase will be applied to increase search efficiency. There will be no language restriction. Table 3 provides the more elaborated search strategy to be applied to PubMed and Embase. The reviewers will also screen the reference list of the studies included in the review for additional eligible studies not retrieved by the search. The searches will be re-run for a final analysis to retrieve the most recent studies eligible for inclusion.

**Table 3:** Search strategy applied to PubMed and Embase.

Terms	
**1 Population**	“Animal Model” [MeSH Terms] OR “Rodentia” [MeSH Terms] OR “Rats” [MeSH Terms] OR “Mice” [MeSH Terms] OR Castor Beaver OR Beavers OR Beaver OR Capybaras OR Capybara OR Hydrochaeris OR Hydrochaeri OR Jerboas OR Jerboa
**2 Intervention**	“selenium” [MeSH Terms] OR “effects of selenium supplementation” OR “levels of selenium” OR “selenium level” OR “selenium supplementation” OR “selenate” OR “selenite” OR “sodium selenate” OR “sodium selenite” [MeSH Terms] OR “selenate doses”
**3 Outcomes**	**“**Blood Glucose” [MeSH Terms] OR “Glucose Tolerance Tests” [MeSH Terms] “Resistance, Insulin” [MeSH Terms] OR “hyperinsulinaemia” OR “glucose metabolism” OR “glucose transport” OR “hyperglycemia” OR “Diabetes Mellitus” [MeSH Terms]
**4 Outcomes**	“glutathione peroxidase” [MeSH Terms] OR “GPx1” OR “glutathione” OR “overexpression of glutathione peroxidase” OR “overexpression GPx” OR “GPx activity” OR “glutathione peroxidase activity”
**5 Filter**	Animal studies
**Equations**	
[Component 1] AND [Component 2] AND [Component 3].	
[Component 1] AND [Component 2] AND [Component 4], include the Component 5.	

### Study selection and data extraction

All articles found by the search strategy will be exported to the Rayyan QCRI application for initial selection by considering their titles, abstracts, and keywords. The full text of the eligible studies will be retrieved for detailed reading. Other studies in the references of the articles included in the review could also be researched.

A standardized form elaborated previously by the reviewers will be used for data extraction. Missing or additional data for a particular study will be requested from the corresponding author via email by the reviewers (maximum two attempts).

**Fig 1:**
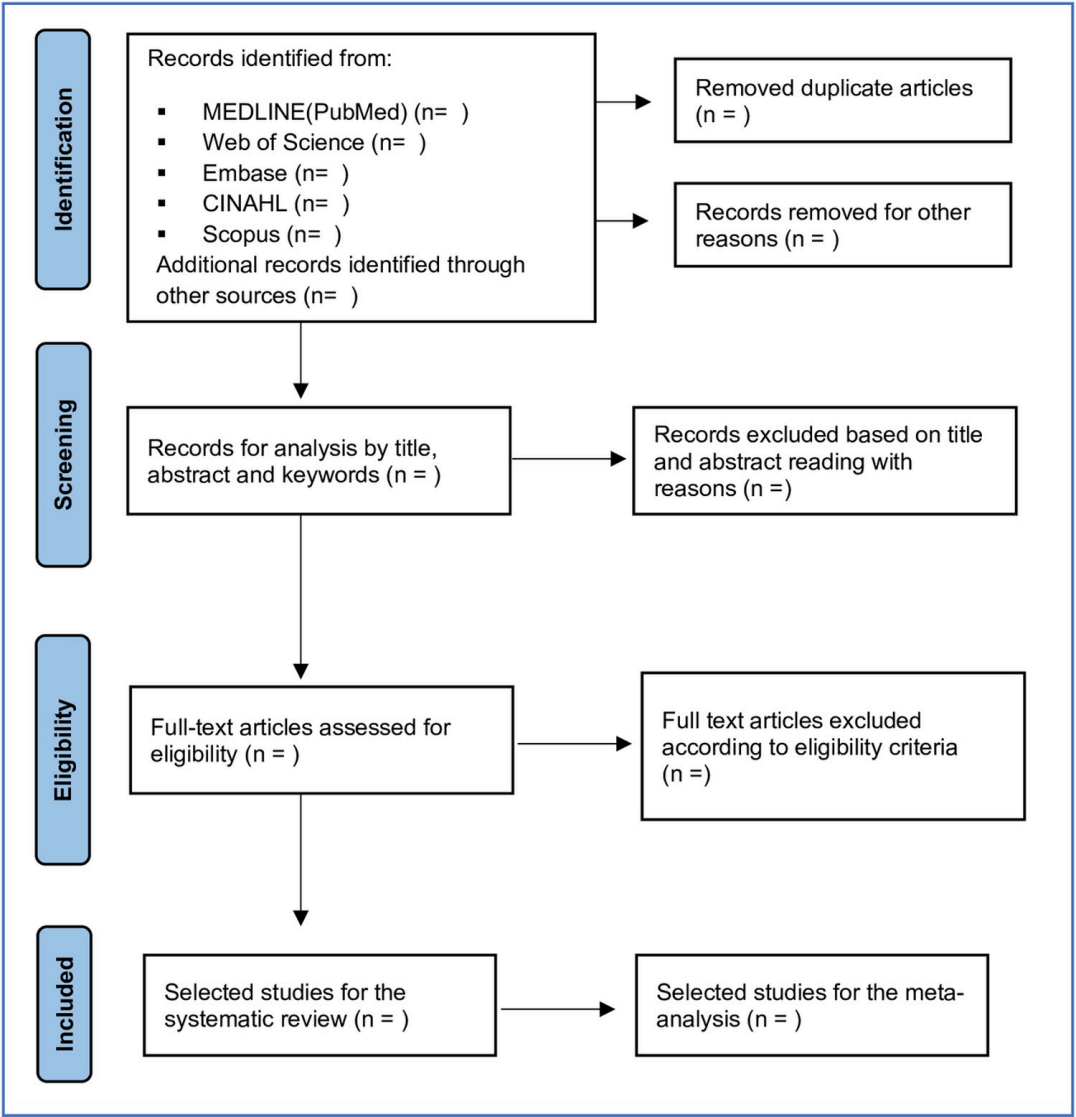
Flowchart for the selection of articles based on PRISMA-P.

The selection of studies and data extraction will be carried out independently by two reviewers (RLF and AWF). Any disagreement between the reviewers over the eligibility of studies will be resolved through discussion and consensus, when no resolution is reached, a third reviewer (RNC) will be involved in the decision. A record of the reasons for excluding studies at all stages of the review will be maintained. The results of the selection or exclusion of studies will be reported using the PRISMA flowchart, as shown in Fig 1.

### Data extraction

Data including first author, year of publication, study ID, original language of publication, sample size, and the forms of supplemented Se will be extracted. In addition to the data described in Table 2, data related to the type of population and intervention will also be extracted, including experimental design, the number of experimental groups and follow up duration, group allocation methods and outcomes, animals’ species used, animal sex, animal age, type of Se supplementation, route of administration, dosage, frequency, time of exposure to supplementation, and a complete description of the basal diet components.

### Risk of bias and quality assessment

The risk of bias for each study included will be assessed by two independent review authors (RLF and RNC) using the Systematic Review Center for Laboratory Animal Experimentation (SYRCLE) risk of bias tool [17]. Each criterion will be assigned high, low, or unclear risk-of-bias values. We will use the Collaborative Approach to Meta-Analysis and Review of Animal Data from Experimental Studies (CAMARADES) [18] checklist since this tool combines the reporting of several measures to reduce bias with indicators of external validity. Each study will be given a quality score out of a possible total of 10 points, and the mean score will be calculated. Studies that score 1–5 will be considered “low quality,” while scores 6–10 will be considered “high quality.” The Grading of Recommendations Assessment, Development, and Evaluation Working Group methodology (GRADE) [19] will be used to classify the certainty of evidence.

#### Strategy for data synthesis

Data from eligible studies will be described in a narrative synthesis following Synthesis Without Meta-analysis (SWiM) reporting guideline [20]. This initial narrative synthesis will summarize study characteristics, population (animals), type of selenium supplementation used, dose, route of administration and comparison studied in textual form.

If a meta-analysis will be performed, using the standardized mean difference (SMD) and 95% confidence interval (CI), calculated by subtracting the mean of the control group from the mean of treatment groups divided by the pooled standard deviation of the two groups, for continuous outcome data. The treatment effect of binary outcomes data will be summarized using risk ratios (RRs) with a 95% confidence interval (CI). A random-effects model will be used to pool the data.

The heterogeneity between the studies will be verified using the Cochrane Q test and quantified by the I2 test, with values >50% representing high heterogeneity. The meta-analysis will be provided when at least two studies match the eligibility criteria of the review.

### Analysis of subgroups or subsets

If sufficient data are available, subgroup analysis will be performed by type of Se supplementation. Sensitivity analysis will be conducted between low and high risk of bias and with or without biased studies. We will conduct a funnel plot and Egger test to check for possible reporting bias if enough studies (at least 10) included in this review are available.

## Discussion

A total of more than 1000 papers dealing with the subject were identified in the databases described in the search strategy of this protocol, reinforcing that experimental, epidemiological, and randomized clinical trials involving the association between excess Se and hyperglycemia are increasingly frequent [21–24]. Even though preclinical studies indicate benefits in animal models, the results are controversial and do not allow the establishment of supplementation strategies, especially in populations of healthy individuals.

The use of medications and nutritional supplements containing Se has increased due to the popularization of the antioxidant and immunological functions of Se [25]. Moreover, the increased availability of these products on the market contributes to self-medication practices by individuals, resulting in excessive Se intake that can culminate in toxicity [26–28].

Considering that healthy adult and those with chronic diseases are increasingly using Se supplements, and clinical trials on the safety of this intervention are still inconclusive, evaluating the efficacy and potentially severe adverse effects in animal models may guide researchers to improve protocols for clinical trials.

Therefore, this review could help fill existing gaps on the action of Se in glucose metabolism, given sufficient evidence on the efficacy of Se supplementation in healthy animal models, guiding the scientific community towards safe decisions in clinical trials.

However, despite adequate protocols, methodological flaws such as lack of randomization, concealment of allocation, lack of blinded outcome evaluation, and high heterogeneity in the doses used in supplementation, as well as among the included studies, can compromise the level of evidence generated in this systematic review.

The doses’ heterogeneity includes the treatment duration, the different vehicles of ingestion (diet, water, isolated supplements), and the chemical forms of Se used, which increases the risk of bias between studies. Organic compounds such as selenomethionine (SeMet), selenocysteine (SeCys), and methyl derivatives are absorbed more quickly, unlike inorganic forms such as selenate (SeO_4_-^2^) and selenite (SeO_3_-^2^) [29]. These mechanisms drive Se bioavailability, and toxicity pathways to different responses in rodents tested in experimental models.

Moreover, different methods of measuring the outcomes, lack of sample calculation, and small samples (“n”) may influence the quality and generate biases in disseminating systematic reviews based on animal experimentation, compromising extrapolation to humans [17, 30]. The authors included the risk of bias assessment by SYRCLE, a validated tool for in vivo studies to mitigate these weaknesses [17].

Thus, the review will be carefully interpreted regarding differences in experimental design, internal validity, quality of evidence, and risk of bias to allow the results of studies based on animal models to support future clinical trials [31].

The results will provide evidence to help in selecting doses, intervention times, and forms of Se to be administered in clinical trials while at the same time elucidating mechanisms of the interaction of excess Se with glycemic control.

## Supporting information

S1 Checklist PRISMA-P 2015 checklist. (DOCX)
